# The Role of Histone Ubiquitination during Spermatogenesis

**DOI:** 10.1155/2014/870695

**Published:** 2014-05-19

**Authors:** Kai Sheng, Xiaotong Liang, Sizhou Huang, Wenming Xu

**Affiliations:** ^1^SCU-CUHK Joint Lab for Reproductive Medicine and Key Laboratory of Obstetric & Gynecologic and Pediatric Diseases and Birth Defects, Ministry of Education, West China Second University Hospital, Sichuan University, Chengdu 610041, China; ^2^Polypeptide Hormone Laboratory, Department of Medicine, McGill University and Research Institute of the McGill University Health Centre, Montreal, QC, Canada H3A 0C7; ^3^Department of Histology and Embryology and Neurobiology, Chengdu Medical College, 601 Tianhui Road, Rongdu Avenue, Sichuan, Chengdu 610083, China

## Abstract

Protein ubiquitin-proteasome (ubiquitin-proteasome) system is the major mechanism responsible for protein degradation in eukaryotic cell. During spermatogenesis, the replacement of histone by protamine is vital for normal sperm formation, which is involved in ubiquitination enzymes expressed in testis. Recently, histone ubiquitin ligases have been shown to play critical roles in several aspects of spermatogenesis, such as meiotic sex chromosome inactivation (MSCI), DNA damage response, and spermiogenesis. In this review, we highlight recent progress in the discovery of several histone ubiquitin ligases and elaborate mechanisms of how these enzymes are involved in these processes through knockout mouse model. Using Huwe1, UBR2, and RNF8 as examples, we emphasized the diverse functions for each enzyme and the broad involvement of these enzymes in every stage, from spermatogonia differentiation and meiotic division to spermiogenesis; thus histone ubiquitin ligases represent a class of enzymes, which play important roles in spermatogenesis through targeting histone for ubiquitination and therefore are involved in transcription regulation, epigenetic modification, and other processes essential for normal gametes formation.

## 1. Introduction

### 1.1. Ubiquitination Proteasome System

Protein ubiquitin-proteasome (ubiquitin-proteasome) system is the major machinery responsible for protein degradation in eukaryotic cell [1, 2]. Ubiquitin is a small protein containing only 76 amino acids that can be attached to the lysine residues of the other proteins for protein ubiquitination. Two major substrate ubiquitination methods exist: addition of a single ubiquitin molecule named monoubiquitination and addition of different types of ubiquitin chains named polyubiquitination [3]. Polyubiquitinated substrate proteins may be degraded by the 26S proteasome when the lysine residue is linked to a plurality of ubiquitin molecules while monoubiquitinated lysine residues may affect the three-dimensional structure of the protein and are involved in the functional regulation of the protein [4, 5]. In general, ubiquitination process requires three enzymes to assume the roles, including the ubiquitin activating enzyme (E1), ubiquitin conjugating enzyme (E2), and the ubiquitin ligase enzyme (E3) [5, 6]. In these three enzymes E3 ligases are considered as the major type with recognition and catalysis of the substrate specificity—with ubiquitin E2 transferred to the role of specific substrates. E3 has a high degree of specificity, which determines the specific role of the different E3 ubiquitin enzymes in different tissues and at a variety of developmental stages [5, 7]. After the tag of E3 ubiquitin ligase, polyubiquitinated substrate will be degraded by 26S proteasomes. 26S proteasome is a 2.5 mDa multisubunit consisting of two 19S regulatory subunits and a 20S catalytic subunit [3, 8]. Ubiquitin proteasome plays a critical role in protein metabolism of the cells because with the inhibition of this system, the degradation of the major proteins in the cells is suppressed [9]. The ubiquitinated substrates could also be reversed by the deubiquitination enzymes, therefore maintaining balance for protein metabolism [10, 11]. Although the major function of ubiquitin ligases is related to protein degradation, ubiquitin has also been shown to be involved in mediating the signaling pathway. For example, the ubiquitination of P53 by MDM2 and Huwe1 is critical for P53 activity and for the apoptosis promoting function [12]. Another emerging observation is that ubiquitin ligases could both activate or inhibit gene expression, depending on the context of the transcription complex [13]. In fact, the ubiquitin process is broadly involved in almost every range of eukaryotic cell functions [5, 14].

## 2. The Histone and Its Posttranslational Modification

Histones are highly basic proteins that bind tightly to the acidic DNA to form chromatin. Five types of histones have been identified: H1, H2A, H2B, H3, and H4. Two molecules each of H2A, H2B, H3, H4, and one linker H1 associate with 147 bp of DNA to form a nucleosome, the fundamental packing unit of the chromatin [15, 16]. Although previously viewed as relatively inert structural proteins, histones are now recognized to be the targets of multiple dynamic posttranslational modifications, particularly on their C-terminal tails. The major ones are methylation, acetylation, phosphorylation, sumoylation, and ubiquitination [16, 17]. Such posttranslational modifications regulate the structure of the chromatin and the recruitment of other proteins. The net effect of these changes is to modulate gene expression. The pattern of these posttranslational modifications is now referred to as the histone code [18]. In general, acetylation and phosphorylation accompany gene transcription, while sumoylation typically mediates transcriptional silencing [19]. Methylation and ubiquitination are implicated in both activation and repression, depending on the context, temporal and spatial arrangement of the modifications.

In this review we focus on the histone ubiquitination, and specifically on the role of histone ubiquitination in spermatogenesis. We summarize the specific roles of histone ubiquitination in key steps, such as MSCI, spermiogenesis, and sperm quality control during spermatogenesis.

## 3. Histone Ubiquitination during Spermatogenesis

Spermatogenesis is a complex process by which primitive, unipotent stem cells divide to either renew themselves or produce daughter cells that become spermatozoa. The normal process begins with spermatogonia differentiation, meiotic division and ends with spermiogenesis, a remarkable morphological transformation process, which includes (1) formation of acrosome, containing hydrolytic enzymes crucial for penetration through the oocyte during fertilization; (2) flagellum formation, which involves the development of microtubules arising from the centrioles of the round spermatid; and (3) cytoplasm reorganization by shedding a large part of its cytoplasm as residual body, which is phagocytosed by sertoli cells. Since many proteins and organelles are degraded during this process, it is not surprising that ubiquitin-proteasome pathway plays critical roles in normal spermatogenesis [20]. For example, ubiquitination of mitochondria has long been thought as one important characteristic of spermatogenesis, possibly to promote the predominantly maternal inheritance of mitochondrial DNA in humans [21]. Since recent studies found that sperm mitochondria is vital in sperm function such as ATP production, calcium homoeostasis, the ubiquitination of mitochondrial could also play critical role in sperm physiology [22]. One significant characteristic of spermatogenesis is that the chromatin structure of spermatogenic cells is changing dramatically, resulting in the formation of a highly compressed structure [23, 24]. Chromatin remodeling is a gradual process continuing from spermatogonia proliferation, meiosis to the final sperm formation stage. During the transition of c-kit negative undifferentiated spermatogonia to kit positive differentiating spermatogonia, an epigenetic switch involving Dnmt3a2 is critical to determine whether spermatogonia self-renew or differentiate [25]. Chromatin modification also provides key binding sites for key meiotic related process such as meiotic recombination, DNA damage repair (DDR), and meiotic sex chromosome inactivation (MSCI). Following meiosis, the canonical histones were gradually replaced by transition protein and eventually substituted by protamine so that the chromatins are tightly packed in the sperm head, although in human approximately 10% of histones are retained in mature sperm [15]. Sperm chromatin is a highly organized, compact structure consisting of DNA and heterogeneous nucleoproteins. Protamine confers a higher order of DNA packaging in sperm than that found in somatic cells; thus the condensed and insoluble nature of the highly condensed sperm chromatin protects the genetic integrity of the paternal genome during its transport through the male and female reproductive tracts. Protamine replacement may also be necessary for silencing the paternal genome and reprogramming the imprinting pattern of the gamete. Two isoforms of protamine, protamine 1 and protamine 2, exist in sperm and the ratio of P1 and P2 is tightly regulated to ensure proper spermatid terminal differentiation; the aberrant ratio is an indicator of infertility [15].

During spermatogenesis, there are nonallelic variants of the major histones that also play crucial roles in different stages of spermatogenesis. For example, histone variants such as TH2B, testis-specific histone H1 are present from spermatogonia to spermatid, while other variants, such as H1t2, are expressed during histone-protamine exchange in round/elongating spermatids. The specific expression may indicate their specific function; for example, a recently discovered H2A.Lap1 has been shown to be loaded onto the chromosomes in round spermatids and suggested to have a role in transcription of MSCI silenced genes [16, 24, 26, 27]. The histone-protamine transition is of vital importance in the process of spermatogenesis since spermatogenesis will be compromised if the transition process was blocked [24]. Histone removal and degradation are regulated, at least partially by the ubiquitin system [15, 24, 28].

The last decade has seen more histone ubiquitin ligases being discovered that play critical roles in different aspects of spermatogenesis (Table 1). It should be noted that other ubiquitin conjugating enzymes such as Ube1, Ube, and Ubc are highly expressed in testis and could also play important role in spermatogenesis, although their functions are not elaborated in the present review due to space constraints [34–36]. Deciphering the function of these enzymes involved in spermatogenesis may contribute to mechanistic insight of the etiology of male infertility. For the convenience of readers, we highlighted three aspects of spermatogenesis to elaborate the specific role that histone ubiquitination may be involved in, although it must be borne in mind that normal spermatogenesis is a continuous process.

### 3.1. Histone Ubiquitination and MSCI

MSCI is the mechanism to prevent unpaired region of X and Y chromosomes from initiating cell death. MSCI is important for the completion of the meiotic process because if MSCI is disturbed, the meiotic prophase will be blocked at pachytene spermatocytes stage. During mice spermatogenesis ubiquitinated H2A (uH2A) localized mainly in the XY bodies of primary pachytene spermatocytes, which is a structure of the pseudoautosomal region with gene transcription silenced and remains repressed from transcription in round spermatids to elongating spermatids stage. With the replacement of histone by protamine, uH2A level increased again, indicating that histone ubiquitination was finely regulated during spermatogenic process and may be involved in several steps of spermatogenesis [37]. The XY body was also associated with a series of specific histone modifications, including dual-methylation of H3K9 histone H3, H4 deacetylation, and H2AX phosphorylation. The specific role of ubiquitination of H2A in MSCI is still unclear. Since ubiquitination of H2A plays conserved role in the evolutionary process of gene silencing (RNAi), it may have a similar role in the process of gene silencing within chromosomal XY body. Monoubiquitination of H2AX also plays critical roles in H2AFX phosphorylation and DNA damage repair in somatic cell, highlighting the possibility that ubiquitination process could also be important for other histone modifications to coordinate the process of MSCI [38].

### 3.2. Histone Ubiquitination and Spermiogenesis

Histone ubiquitination regulation and histone acetylation, methylation, and phosphorylation modification are closely related during spermiogenesis. In recent years, studies have found that histone acetylation can undermine the stability of the nucleosome core particle [39]. During spermiogenesis histone ubiquitination may affect histone acetylation process by reducing H2A-H2B dimers free energy to promote transcription. Therefore, there may be coordination for histone acetylation and ubiquitination in the transcription process; a recently described RNF8 gene knockout mice model has confirmed this hypothesis [39]. RNF8 knockout mice testis shows inhibited histone ubiquitination and abolished H4K16 acetylation; a histone modification marks the initial step of nucleosome removal; therefore, the histone ubiquitination and acetylation are highly interactive processes [39]. Ubiquitination of histone and methylation may also have links as it has been well defined in yeast study. Monoubiquitination of histone H2B in yeast leads to methylation of histone H3, which in turn causes telomeric gene silencing [40, 41]. The ubiquitination of H2B controls the binding of the COMPASS complex, which is required for methyltransferase activity in vivo [42]. But the exact mechanisms still require further experimental clarification.

### 3.3. Histone Modification and DNA Damage: Relevance for Sperm Quality and Embryo Development

During meiotic division DNA damage repair is a unique process, which not only shared the somatic counterpart but also evolved sophisticated regulation to ensure genetic integrity and diversity to be passed into offspring. Recent studies suggest that epigenetic modification of proteins and DNA in the sperm production process is very important for early embryonic development; aberrant change of these epigenetic modifications will lead to male infertility [43].

How does the H2A ubiquitination process affect DNA damage repair? One possibility is that H2A ubiquitination sites may promote the recruitment and stability of the adjustment factors such as RNF8s through ubiquitination mechanism [44]; another possibility is that uH2A could possibly affect other histone modifications, such as H2B and H3 hypermethylation [39]. Although the mechanism is still elusive, studies have shown that sperm epigenetic modifications such as DNA demethylation, miRNA expression, and histone modification play critical roles in spermatogenesis; furthermore, they share similarity with the characteristics of embryonic stem cells. Thus, de Boer et al. proposed that the sperm maybe the source of genetic instability across generations (transgeneration) and mutation (epimutation) source of offspring [45]. Applying ChIP on ChIPtechnology, Carrell also found that in human sperm histone modifications are enriched in Hox developmental genes, which may be very important for early embryonic development [15], further confirming the importance of histone modifications for the early development.

It has been shown that high levels of H2B can cause DNA damage [46], and chromatin structure changes will affect the stability of the genome, inducing the mammalian genome DNA double-strand breaks (DSB) and causing phosphorylation of isomers of histone H2A139 serine locus [46]. This modification is called *γ*H2AX. *γ*H2AX has become a sensitive indicator of DNA DSB formation [47]. Recent work shows that *γ*H2AX detection may be a better indicator for monitoring the development of cancer genetic variation associated with tumor progression [48]. Nevertheless, histone ubiquitin ligase has been shown affecting DNA damage through degradation of key DDR enzymes and therefore the function is linked to sperm quality; the deregulation of its expression is relevant to the infertile patients with high DNA damage sperm [43]. Histone ubiquitination mediated by RNF8 suppresses more homologous recombination and promotes nonhomologous end joining recombination, which are shared with similar mechanism during meiotic division; therefore, RNF8 is important for both meiotic DDR and recombination during spermatogenesis [43, 49, 50]. Furthermore, ubiquitin process is a reversible process and could be reversed by the deubiquitinating enzyme, which has also been shown to be important for spermatogenesis [51, 52]. Whether deubiquitinating enzyme can affect DNA damage in the process of spermatogenesis and its underlying mechanism remains unclear [53]. Interestingly, a recent paper shows that sperm proteasome could be the candidate ZP receptor ubiquitin ligase during fertilization process, thus playing important roles in fertilization process [54, 55].

Although only about 10% of the sperm chromatin is retained with histone, the histone retention and modification are crucial for genome reprogramming during embryo development. As both the repressive and active histone methylation are marked as distinctive promoters of human and mouse sperm [56], sperm histone modifications could be the critical code involved in transcriptional regulation important for embryo development, and the aberrant expression could be related to the embryo defects or miscarriage disease [15, 45]. Gill et al. proposed a model of epigenetic inheritance by nucleosomes between generations [57], in which “intrinsic intergenerational/transgenerational inheritance program” was proposed to explain the observations that prepatterning of histone modifications and RNA-based inheritance in gametes could be inherited over successive generations [58, 59]. The following are several candidate ubiquitin ligases which have shown histone ubiquitin activity either in vitro and in vivo.

## 4. Representative Histone Ubiquitin Ligases and Their Roles in Spermatogenesis

*Huwe1.* Huwe1 also known as Lasu, Mule1, and ARF-BP, is a UBA, WWE, BH3, and HECT domain containing protein, and its molecular weight is about 483 kDa [60]. Despite the facts that HUWE1 has been shown to be highly expressed in several types of tumors, and the degradation of substrates includes several key genes, such as p53, c-myc related to cell proliferation and transformation, its exact function in the body is still unclear [12, 60, 61]. Additionally, it has also been found that Huwe1 can bind to and degrade topoisomerase protein 1 (TopBP1), ATR (ataxia telangiectasia and RAD3-related protein), and so forth, thereby adjusting DNA base-pair repair after UV radiation [60, 62].

Huwe1 is expressed in a broad range of tissues. Interestingly except in the neuron and testicular tissues in which it is expressed in nuclei, in all the other somatic tissues the expression is cytoplasmic, suggesting that its function maybe diversive in different tissues. In testis, Huwe1 is mainly expressed in the nucleus of spermatogonia and pachytene primary spermatocytes [30, 36, 60]. In vitro experiments show that Huwe1 can degrade testes histones, H2A, H2B; therefore, it has been hypothesized to play an important role in the early spermatogonia chromatin modification process [30]. In vitro studies on mice also found that Huwe1 can bind to E2 enzyme UBC4 and degrade histone [30]. Since ubiquitination of H2A is carried out in the XY body, it has been hypothesized that Huwe1 is possibly involved in chromosome recombinant and DNA repair process, thus playing a very important role in the proliferation and differentiation of spermatogonia, as well as the regulation of sex chromosomes inactivation during meiosis. 

*UBR2.* The UBR family belongs to ubiquitin E3 ligase which contains components of the N-end rule proteolytic pathway as well as RING domain. N-end rule proteolytic pathway includes recognition components which recognize destabilizing N-terminal residues of short-lived proteins and has at least three components to guide the substrate in accordance with the hydrolysis of the N-end rule [37, 63, 64]. In mammals there are two homologues, UBR1 and UBR2 homologues, and recently two additional proteins, termed UBR4 and UBR5, were found to share similar binding to certain destabilizing N-terminal residues [31]. Mice UBR1 and UBR2 have a 200 kDa overlapping sequence (47% identity and 48% similarity) [65, 66]. Knockout of UBR2 mice UBR2 (−/−) showed phenotypic manifestations of embryonic lethality. Interestingly, most female pups have died in the embryonic period, while most UBR2 (−/−) knockout male mice can survive and appear infertile, very likely because of postnatal testicular degeneration. UBR2 (−/−) mice testes have approximate normal morphology and the spermatogonia were intact; however, the spermatocytes arrest in leptotene/zygotene and pachytene stage and eventually lead to apoptosis. The most significant defects of UBR2 (−/−) mice show spermatocytes without complete synaptonemal complex. The UBR protein could mediate histone ubiquitination [37]. UBR2 is positioned in meiotic chromatin regions, including the axial chromosome inactivation region, and regulates transcriptional silencing by regulating H2A ubiquitination. UBR2 is linked to E2 HR6B, with its substrate H2A. Specific UBR2 and ubiquitination of H2A (uH2A) mark meiotic chromatin regions to transcriptional silencing. In the spermatocytes of UBR2 mutant mice H2A ubiquitination cannot be induced during meiosis, which seriously affects the chromosome-wide transcriptional silencing of genes linked to unsynapsed axes of the X- and Y-chromosomes. The infertility likely results from UBR2 histone ubiquitination insufficiency triggering the pachytene checkpoint system.

Yang and his research team also found that UBR2 ubiquitin ligase has another function except for the existing role of mediating protein degradation. UBR2 forms a stable complex with Tex19.1 protein. Tex19.1 is a germ cell-specific expressed protein [63], and mutation of Tex19.1 in mice the spermatogenesis will be seriously affected. The interaction of Tex19.1 with UBR2 is stable and is not related to N-end rule pathway; thus, it is unlikely that UBR2 is a ubiquitin ligase for Tex19.1. The phenotype of Tex19.1 knockout mice is similar to Ubr2 deficient mice in three aspects: meiotic defects (heterogeneity of spermatogenic defects); asynapsis of synaptonemal complex during meiosis and embryonic death preferentially affecting females. In the germ cells of the UBR2 defects, Tex19.1 is transcribed, but lack the translation of protein, implying the binding of UBR2 to Tex19.1, could affect the stability of latter.

*RNF8 and RNF168.* RNF8 is a polypeptide consisting of 485 amino acids, the N-terminal contains RING finger domain, C terminal includes FHA domain, and these two domains are indispensable for its ubiquitin ligase activity. Like other RING finger domains containing protein, RNF8 has ubiquitin E3 ligase activity [49]. RNF8 can bind to phosphorylated MDC1, a critical mediator of the mammalian DNA damage checkpoint and enriched in the DNA damage site, activate a series of ubiquitination processes, recruit RAP80-BRCA1 complex in the DNA damage site, and bind to 53BP1 to set check point for DNA damage response and promoting cell survival [32, 43]. Mouse model study indicated that mutation of RNF8 will compromise normal spermatogenesis, as well as showing increased sensitivity to ionizing radiation. Reorganization of chromatin is thought to compact the parental genome to avoid damage of DNA in the sperm head. Histone ubiquitination is also linked to the sex chromosome inactivation meiotic sex chromosome inactivation (MSCI). RNF8-dependent ubiquitination can also induce histone H4K16 acetylation, which is the first step in the migrating nucleosome [39]. A recent study shows that RNF8 could regulate escaping gene activation from MSCI in spermatid and therefore provide connections for a DNA damage response factor and epigenetic activation during spermatogenesis, highlighting the functional interaction between MSCI and DNA damage response and epigenetic modification [67].

RNF168 is another RING-type ubiquitin ligase that also works with the UBC13 E2 enzyme. Mutations of RNF168 are associated with the RIDDLE syndrome, a rare genetic disease characterized by cellular defects in repairing DSBs. Knockout of rnf168, another ubiquitin ligase targeting histone, could also impair spermatogenesis [33], Recent studies suggested a possible two-step model for RNF168 recruitment to DSBs: after DNA damage RNF168 is initially recruited and ubiquitinates H2A and H2AX with RNF8-dependant pathway, followed by association to DSB-flanking chromatin with K63-linked ubiquitin chains conjugated by both RNF8 and RNF168. RNF8 and RNF168 in DSB-flanking chromatin could also serve as binding sites for the recruitment of the downstream effectors of the DDR pathway such as BRCA1 and 53BP1. Thus the RNF8 and RNF168 interaction could play critical roles in both spermatogenesis and DNA damage response in germ cell and somatic cell, respectively [33].

## 5. Conclusion

**Figure 1:**
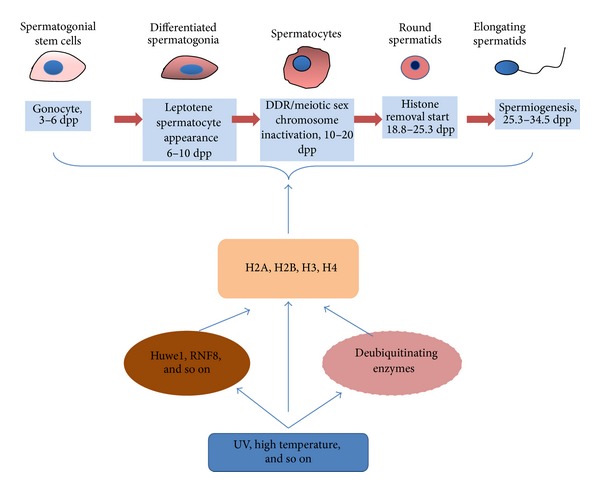
Indicated is the timeline of mouse spermatogenesis. Major stages of spermatogenesis, and cell types were shown in the diagram. Major ubiquitin ligases, including Huwe1, RNF8, were expressed in different stages of spermatogenesis. Histone ubiquitin ligase plays important roles in ubiquitination of histone and its variants, thus implicating in diverse stages of spermatogenesis. Dpp means postnatal days to show development stage of normal spermatogenesis. From 3 to 6 days, gonocyte is the major type in seminiferous tube, from postnatal 6 (6 dpp), leptotene spermatocyte appears, while during 10 dpp to 20 dpp, DDR and MSCI were observed in spermatocyte, histone removal begins from 18.8 dpp to the final stage; spermiogenesis begins from 25.3 to 34.5 dpp with drastic morphology change to form the mature sperm.

In conclusion, the importance of histone ubiquitination has recently been established and the mechanism has begun to be elucidated. Therefore the coming years could see more histone ubiquitin ligases awaiting to be discovered and their specific mechanisms need urgent clarification. Nevertheless, histone ubiquitination might not account for the only mechanism of the degradation of histone during spermatogenesis. For example, a recent paper shows that a testis enriched in proteasomes activator PA200 is required for acetylation-dependant as well as polyubiquitin-independent degradation of histone, thus playing critical roles during DNA double-strand breaks and spermatogenesis [68]. It should be noted that ubiquitination of histone can be reversed by deubiquitinating enzymes. USP enzyme has also been shown to play critical roles in spermatogenesis and mutation/SNP of the USP enzyme could result in male infertility [51, 52]. We hypothesized that Huwe1 and other ubiquitin ligases could be involved in the diverse aspects of spermatogenesis through histone ubiquitination and regulate histone modification (Figure 1). It can be seen that during spermatogenesis the histone experiences a dynamic modification process; under normal circumstances, this regulation is related to a variety of physiological processes, like sex chromosome inactivation, DNA damage repair, and so forth. Environmental stimuli, such as high temperature, chemical substances could also lead to histone modification abnormalities and affect the regulatory pathways during spermatogenesis, leading to the abnormal development of the zygote, including the impact of early gene mutations and chromosomal abnormalities [69, 70]; in the same time, they may also cause genomic instability for zygotes and somatic cells, which is closely related to development defects and individual cancer susceptibility [23, 33, 71]. Histone ubiquitin ligases such as Huwe1 could also play roles in regulation of genome stability, as it has been shown that Huwe1 is required for apoptosis response by targeting other histone modification enzymes such as HDAC2 [72]. The study for histone ubiquitin ligase shall not only shed new light on the mechanism of epigenetic phenomenon during spermatogenesis, such as chromosome inactivation of sex chromosome and DNA damage repair process and their effects on sperm production problems; it will also clarify to what extent the epigenetic modification could impact the health of the next generation.

## Figures and Tables

**Table 1:** Current known histone ubiquitin liagses with KO mice model.

Histone Ubiquitin Enzyme	Target	Blocking stage	KO phenotype
HR6B		Spermatid	Infertile [29]
UBC4-t		Spermatocyte	Fertile [30]

Huwe1	H2A, H2B, H3, H4	Spermatogonia	Infertile (unpublished data)
UBR2	H2A	Meiosis	Infertile [31]
RNF8	H2A, H2B	Spermatocyte spermatid	Infertile [32]
Rnf168	H2A	Meiosis	Infertile [33]

## Abbreviations

DDR: DNA damage response
RAP80: Receptor-associated protein 8080
53BP: p53 binding protein 1
USP: Ubiquitin-specific protease.
